# Effects of the hippocampus on the motor expression of augmented breaths

**DOI:** 10.1371/journal.pone.0183619

**Published:** 2017-08-23

**Authors:** Itopa E. Ajayi, Paul C. Mills

**Affiliations:** The University of Queensland, School of Veterinary Science, Queensland, Australia; National Yang-Ming University, TAIWAN

## Abstract

Augmented breaths, also known as sighs, constitute the normal repertoire of breathing in freely behaving humans and animals. The breaths are believed to be generated by neurones in the preBötzinger complex but under modulatory influence from higher brain centres, particularly in the limbic system due to the strong correlations between the expression of emotional behaviours such as anxiety and the occurrence of augmented breaths. The current study examines the role of the hippocampus in the motor expression of augmented breaths, and also examines the characteristics of eupneic breaths surrounding a sigh before and after stimulating the hippocampus in urethane anaesthetised Sprague-Dawley rats. Neurochemical microstimulation using the excitatory amino acid, D,L-Homocysteic acid, was used to locate areas in the hippocampus with the potential to modulated the motor expression of augmented breaths. The CA1 neurone cluster of the ventral hippocampus was found to completely suppress the expression of augmented breaths without affecting the intrinsic properties of the breaths. A similar neurone cluster, but in the dorsal field of the hippocampus, was also investigated and found to have no effects over the expression of augmented breaths. The data supports the hypothesis that there is a structural or functional relationship between neurones of the ventral hippocampus and brainstem nuclei that control augmented breaths. The implications of these findings in the context of behaviours are discussed but with due consideration of experimental conditions.

## Introduction

Eupneic breathing in mammals is broadly divided into two phases, inspiration and expiration, which occur at fairly regular intervals. This breathing rhythm is periodically interrupted by large inspiratory efforts characterised by significant increases in tidal volume, inspiratory duration and diaphragmatic activity. Such breaths are called augmented breaths, and they constitute the normal repertoire of breathing in freely behaving humans and animals [1, 2]. Augmented breaths, also known as sighs, have a biphasic inspiratory curve: The first phase appears like the preceding eupneic inspiratory curves and the second (augmented) phase appears as a gasp or fast inspiration beginning at the peak or near the peak of the first phase [1, 3].

The specific mechanism eliciting augmented breaths is uncertain, but there is evidence that respiratory-related neurones in the ventrolateral medulla, specifically the preBötzinger complex (preBötC), may be responsible for producing augmented breaths [4, 5]. PreBötC activity is modulated by commands from the periaqueductal gray resulting in changes in respiratory patterning [6, 7]. These brainstem structures may form teminal points of limbic ciruits that are activated during the expression of emotional behaviours [5]. However, the source of the neuronal inputs from the limbic system capable of influencing respiratory patterns, particularly the expression of augmented breaths, have not been examined. Thus, the current understanding of the limbic-brainstem circuit physiology involved in the motor expression of augmented breaths is limited, with more research being done on the ascending sensory triggers eliciting sighs.

Eliciting augmented breaths may be dependent upon peripheral sensory triggers, including both mechano- and chemosensory afferent fibres innervating the respiratory system. For example, augmented breaths are believed to prevent atelectasis [8], which is a partial collapse of the lung air sacs due to progressively insufficient lung inflations during regular cyclical breathing, perhaps due to enhanced mechanosensory activity related to reduced pulmonary compliance. Alternatively, in rats, hypoxia and hypocapnia increases the frequency of occurrence of augmented breaths [9–11], consistent with chemosensory afferent activation playing a considerable role. A similar increase in augmented breaths was demonstrated when the same peripheral chemoreceptors and mechanoreceptors were directly stimulated with sulfur dioxide (SO_2_) [12], while transection of the vagus, which contains the axons of most pulmonary sensory afferents that feedback to brainstem respiratory control centres, prevented augmented breaths [12, 13]. These studies reinforce the involvement of peripheral afferent nerves in the regulation of augmented breaths.

However, other investigations have proposed the existence of central modulators or descending motor mechanisms originating from higher brain regions as a principal determinant of augmented breaths. For instance, Shahan et al. (2004) [14] reported that activation of discrete neurone populations in the dorsal PAG increased the frequency of augmented breaths. Disinhibition of the medial hypothalamus has also been reported to increase the frequency of augmented breaths [15]. The PAG and hypothalamus can be considered as relays between the limbic and autonomic nervous systems and may, therefore, receive modulatory signals from forebrain structures that provide the primary drive for augmented breath regulation. In this regard, augmented breaths maybe tightly regulated with respect to changes in emotions [16, 17]. However, to date, investigations of the role of the limbic system in the occurrence of augmented breaths have not been exhausted.

Within the limbic system, the hippocampus is a potential central modulator of augmented breaths due to its involvement in the emotional expression of stress, anxiety and defensive emotional postures, behaviours shown to affect the frequency of augmented breaths [18]. Consistent with this, our previous study demonstrated modulation of respiratory function by the ventral hippocampus, suggesting that the ventral hippocampus may play a role in the physiological changes required to cope when transiting from one emotional state to another, a factor proposed to affect the expression of augmented breaths [6]. It is, therefore, possible that the hippocampus may affect the occurrence or phase timing of augmented breaths [12]. Thus, the current study examines the role of the hippocampus in the motor expression of augmented breaths, and also assesses the relationship between augmented breaths and cardiovascular function after stimulation of the hippocampus. The study aimed to characterise augmented breaths to a finer detail than has been previously described [1, 3], by assessing breathing characteristics in pre-and post-augmented breath periods as well as during augmented breaths, and comparing this with eupneic breathing (control) in spontaneously breathing anaesthetised animals.

## Methodology

### Animals

The University of Queensland Institutional Animal Ethics Committee, Australia, approved the experiments conducted in this study. Sprague-Dawley rats (n = 16; 350–600 g) of either sex were used for the study. Prior to surgery, animals were housed, by sex, in standard plastic cages under 12:12 hour light: dark cycle and fed water and food *ad libitum*.

### Anaesthesia and surgery

Urethane (ethyl carbamate), a long-acting anaesthetic, at a dose of 1.5 g/kg of body weight was administered intraperitoneally (IP). The depth of anaesthesia was frequently monitored by checking corneal and hind paw-pinch stretch reflexes. Animals were considered to be in a suitable plane of surgical anaesthesia if they were non-responsive to these stimuli. Prophylactic doses of atropine methyl nitrate (0.05 mg/100 g) were also administered intramuscularly (IM) before surgery to reduce respiratory secretions. Femoral artery and venous cannulation were routinely performed to connect the rat to blood pressure transducers and replacement fluid line, respectively. However, fluid therapy was rarely used. Supplementary doses of urethane (0·15–0·3 g/kg) were administered intravenously (IV) through the femoral vein during experiments, as required. During experiments, a good surgical plane of anaesthesia was confirmed by regular breathing and a stable mean arterial blood pressure of 100 ± 20 mmHg even after applying nociceptive stimuli such as paw or tail pinch.

#### Chest wall displacement

A force transducer (WPI, Inc) was used to monitor chest wall displacement. The force transducer works on the principles of measuring static and dynamic tensile loads. A string of stainless steel wire (0.003″ bare, 0.0055″ coated; A-M Systems) was anchored to the loose skin on the dorsum of the thoracic cavity and then attached to the transducer, which was mounted on the stereotaxic frame. This method is preferred over diaphragmatic electromyogram and intra-tracheal pressure recordings because of the clear separation of the phases of inspiration reflected in the tracings of a force transducer. The force transducer gives accurate information of the duration of each component of respiration. However, the accuracy of the force transducer was further validated by electromyogram (EMG) from the crural diaphragmatic activity, where bursts of muscle activity represent inspiration.

#### Diaphragm EMG

**Fig 1:**
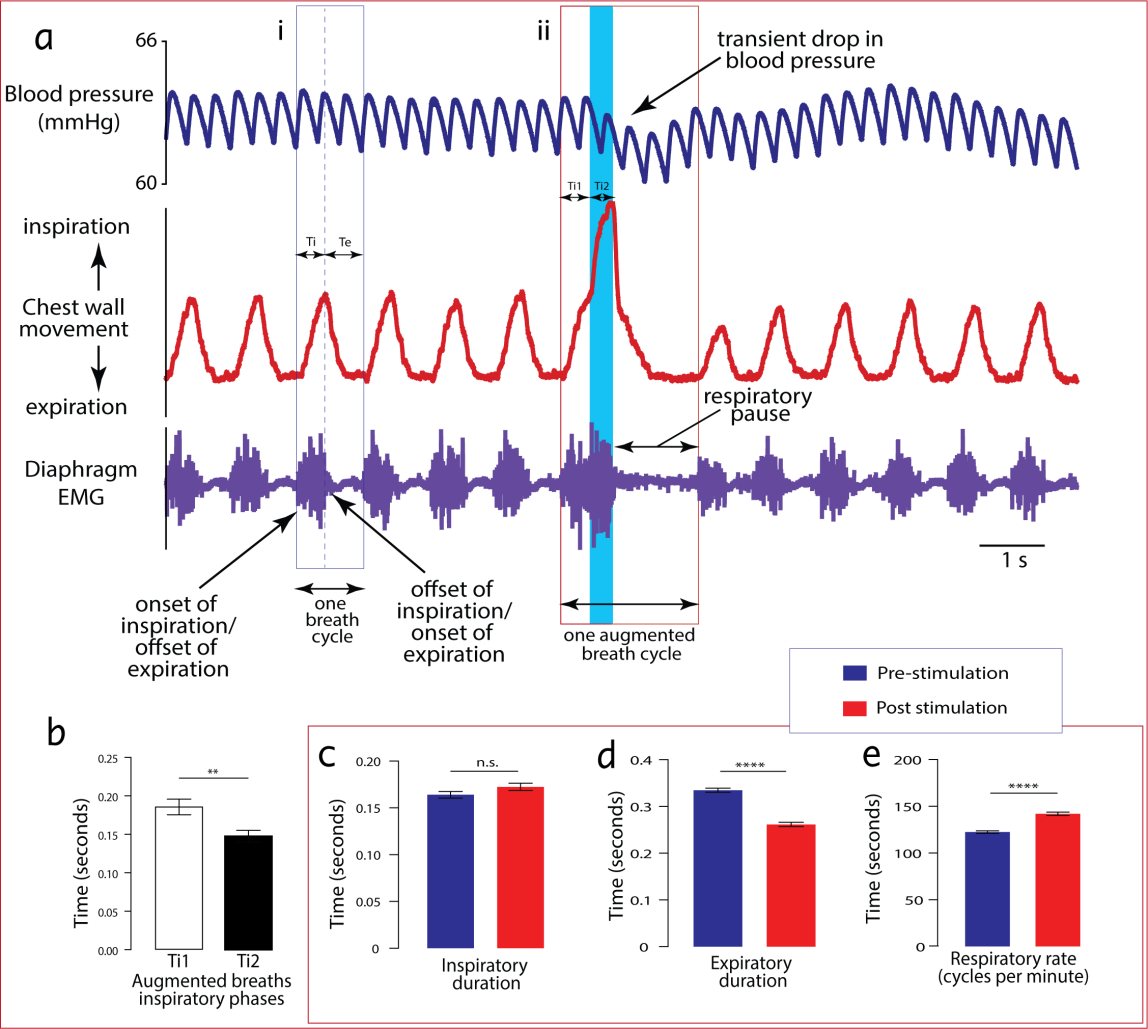
The characteristics of an augmented breath are distinct in chest wall movement tracings, and synchronise with diaphragmatic muscular activity. The figure represents tracings showing all the characteristics of a typical augmented breath cycle (ii) in comparison with a normal breath cycle (i). The middle tracing is a representation of the subjects’ chest wall movement derived from a forced pressure transducer while the lower tracing is the corresponding diaphragm EMG in real time. The inspiratory duration (Ti) represents the entire period of diaphragmatic firing. This period corresponds with the upward curve on the force transducer tracing. However, there are two phases of an augmented inspiratory effort as clearly shown in the force transducer tracing. These phases are labelled Ti1 and Ti2, representing the first and second periods of an augmented inspiratory effort, respectively. Expiration, which is a passive process, requires minimal muscular activity. Thus, the period of no diaphragmatic activity represents the expiratory period (Te), which corresponds with the downward curve on the forced transducer tracing. The bar chart (b) compares the difference between the two phases of inspiration in an augmented breath (n = 30, *P* < 0.05). The bar charts (c–d) summarise the properties of five breathing cycles before (blue bars) and five breathing cycles after (red bars) an augmented breath.

In each animal, a transverse incision was made through the *rectus abdominis* muscle at the level of the xiphoid cartilage. The liver was then reflected to expose the crural diaphragm. A pair of fine platinum wire electrodes was implanted in the crural diaphragm (0.003″ bare, 0.0055″ coated; A-M Systems). Electrode lengths of about 20 cm were used for each recording. A portion of the Teflon coating was stripped at the end and middle of the electrodes (approximately 5 mm each). The electrodes were then threaded through the crural diaphragm by secure attachment to a 17 mm ½ circle reverse cutting suture needle. The electrodes were pulled through until the bare middle portion was in contact with the diaphragm. The loop was knotted to maintain contact and so that it was not easily pulled out of the muscle. Caution was applied to prevent pneumothorax. The stripped end was then connected to a bio-amplifier through a wire relay. A reference electrode was attached to the body. The abdominal incision was closed with a continuous suture pattern, holding both the muscles and skin together to maintain contractile pressure. Skin adhesives were also applied to secure the stitch. At the end of each experiment, the diaphragm was carefully removed to assess electrode placement. Diaphragmatic EMG during an augmented breath shows prolonged muscular activity during inspiration with peak amplitudes higher than normal breaths [19]. Also, the EMG tracing clearly shows a period of respiratory pause after each augmented breath (Fig 1). Coordination of both methods, forced transducer and DEMG, was used to enhance precision in calculating the duration of inspiration and expiration. A heating pad was used to maintain the body temperature between 37–38 ^o^C.

Inspiratory duration (Ti): Each burst of diaphragmatic electromyogram activity was considered an inspiration. The diaphragm is mostly active during inspiration and passive during expiration. Thus, the start of diaphragmatic muscle firing, as captured by the EMG tracing (Fig 1), was indicative of the start of inspiration and the entire period of firing was defined as the inspiratory duration (Ti). The precise duration was calculated offline by identifying such periods of activity in the LabChart software and obtaining its numerical value in seconds from the ‘Data Pad’ function of the same software.

Expiratory duration (Te): The duration from the end of an inspiratory period to the start of the next was defined as expiratory duration. This period was extracted following the same procedure used to extract Ti.

### Craniotomy and microinjections

Animals were placed in a prone position and carefully fitted in a stereotaxic apparatus (Narishige Scientific Instrument Lab.). An incisor and ear bars was used to firmly anchor the head. Protective ophthalmic ointment (GenTeal ®) was applied, and the scalp was shaved and disinfected with an antiseptic solution (Betadine®) or 70% ethanol solution. A midline incision of about 2 cm was then made over the scalp using a scalpel blade or cauterizer (Erbe ICC 200) to minimise bleeding while exposing the skull. Bregma and lambda were identified and used to ensure that the skull was in a flat position by adjusting them to the same stereotaxic position. Small boreholes (≈ 2 mm in diameter) were drilled using a craniotomy drill (Microtorque II, SDR Scientific) to expose and allow access to the brain. A nick was made in the dura mater to enhance precision and prevent destroying the fragile tip of glass micropipettes. Glass micropipettes with tip diameters of 20–30 μm were pulled using a Sutter pipette puller (P-97, Novato, CA). The micropipettes were calibrated and coupled to Hamilton syringes to enable delivery of precise volumes of drugs into the brain. Coordinates of the anterior-posterior position, medial-lateral position as well as the depth of Bregma were recorded, and calculations were made for various target regions according to Paxinos and Watson, 2007 [20]. The ventral hippocampus was chemically mapped with microinjections of the excitatory amino acid, D, L-Homocysteic acid (DLH, 50 mM, 200–400 nL) until areas that have influence over the expression of augmented breaths were identified. The dorsal hippocampus was also examined for control and comparison. Quantitative analyses of the typical characteristics of augmented breaths were analysed using 30 representative augmented breaths selected randomly from six animals, selected mostly during baseline breathing periods. Tracings of augmented breaths that were distorted by external factors such as adjusting the animals’ position during experimental/surgical manipulations of were excluded. The breaths surrounding each augmented breath were also assessed. For this, the five breaths each immediately preceding and succeeding an augmented were analysed for both inspiratory and expiratory duration. The rats were spontaneously breathing room air throughout the period of the experiment, and in a prone position.

### Tissue sectioning and histochemical staining

At the completion of each experiment, rats were euthanased with an intravenous injection of Nembutal (100 mg/kg), the chest was surgically opened, and the brain perfused through the left ventricle with approximately 300 mL of 0.1 M PBS followed by 300 mL of 4% PFA. The brain was then carefully extracted and fixed in 4% buffered formalin solution for 5–16 hours in a fridge at 4 ^o^C. The brains were then transferred to phosphate buffered saline and stores in the fridge (4 ^o^C) until the time of sectioning. Serial sections were cut at 50 μm using a vibrotome (Zeiss Hyrax V50) and immediately mounted on gelatin-coated slides. Sections were then dehydrated through a graded series of alcohol (50%, 70% 90%, 95%, 100% and 100% for 2 minutes each) and cleared in xylene (2 x 2 minutes). The sections were rehydrated through a descending series of alcohol (100%, 95%, 90%, 70% and 50%), then into distilled water before staining. After staining the sections were dehydrated again (50%, 70% 90%, 95%, 100% and 100% for 2 minutes each), cleared with xylene (2 x 2 minutes) and then cover-slipped with a mountant, depex or fluoromount (Gurr®).

**Fig 2:**
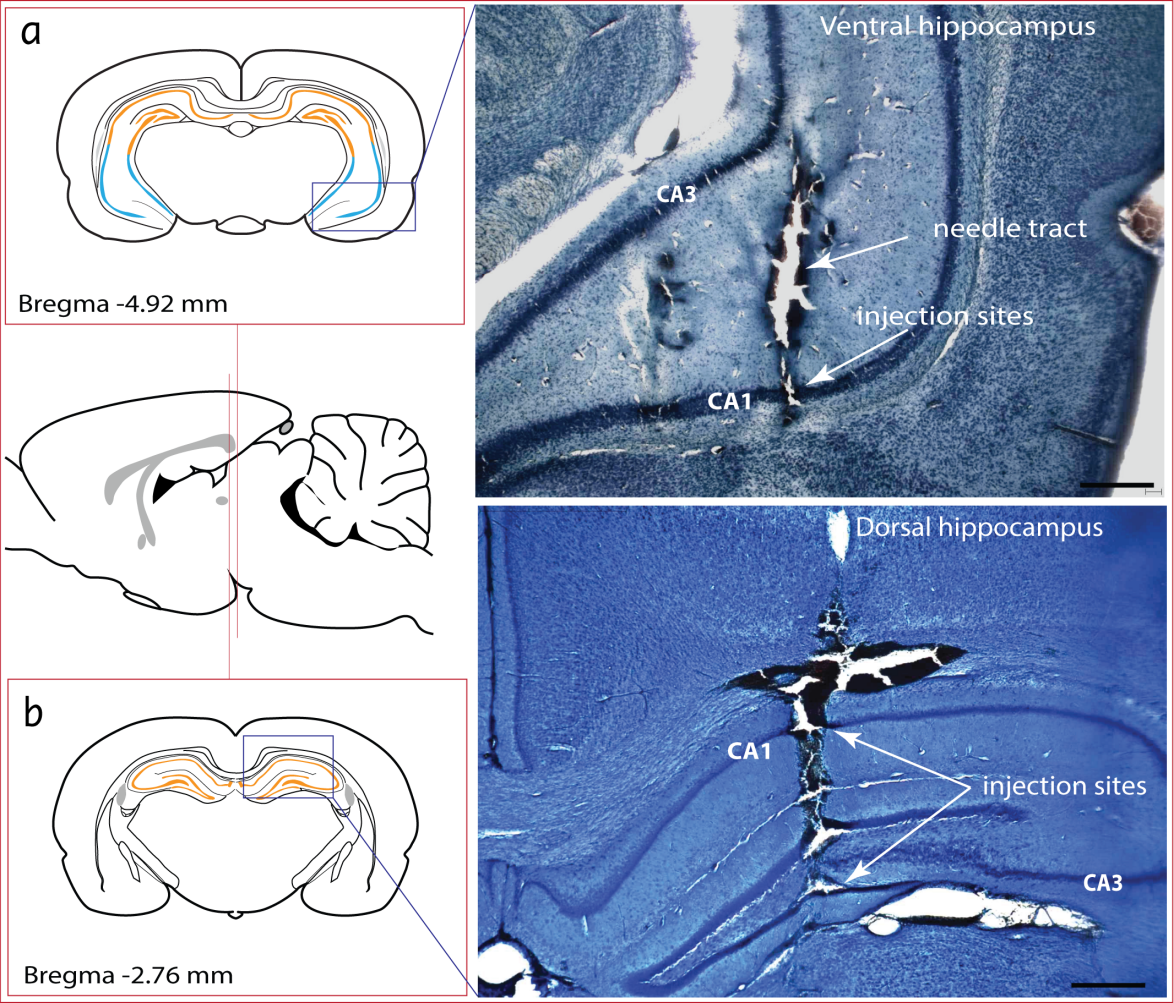
**Representative photomicrographs of stimulation sites in the ventral (a) and dorsal (b) hippocampus**. The diagrammatic illustrations used to indicate the coordinates relative to Bregma were derived from an atlas of the rats’ brain by Paxinos and Watson [20]. Scale bar: 200 μm.

A modified Thionin histochemical staining protocol was routinely used to process brain slices for the visualisation of stimulation sites. Thionin is a strongly metachromatic dye, useful for staining of acid mucopolysaccharides. It is a common nuclear stain and can be used for the demonstration of Nissl substance in nerve cells of the CNS. Thionin at a concentration of 0.1% was constituted from two separate solutions: Firstly, 0.2% Sodium acetate buffer was constituted by adding 1.64 g of sodium acetate anhydrous to 20 mL distilled water and stirring until completely dissolved. Glacial acetic acid (20 mL) was then added to 0.2% sodium acetate buffer. The second solution (0.1% Thionin solution) was made by adding 0.8 g of thionin to the initially constituted 0.2% sodium acetate/acetic acid buffer. Distilled water (760 mL) was then added to the solution and stirred until completely dissolved. The final solution, which efficiently penetrates neurones fixed in 4% paraformaldehyde, was used to stain Nissl substances purple/dark blue while neurones and cell nuclei are purple/blue. Caution was observed not to fix the tissues for too long so that penetration of thionin was efficient. Representative photomicrographs of microinjection sites in the dorsal and ventral hippocampus are presented in Fig 2. A total of 14 injection were properly placed (eight in the ventral hippocampus and six in the dorsal hippocampus) while 2 injection placements were unsuccessful. Hippocampal injections that did not generate any physiological response were not included.

### Data analysis

Respiratory and cardiovascular data were collected for periods of 10 minutes before stimulation (baseline) and at least 30 minutes post DLH microinjection (effect). Data was collected in real time using a Powerlab system and analysed offline. For each augmented breath, the duration of each phase of inspiration (Ti1 and Ti2) was calculated. These periods were compared and plotted using paired Student’s *t*-test on Prism 6 for Mac OS X. The duration of expiration or period of respiratory pause (see Fig 1 for illustration) was also calculated and compared with the expiratory period of eupneic breaths using paired Student’s *t*-test. All values were recorded as mean ± standard error of the mean. A Stereotaxic atlas of the rats’ brain by Paxinos and Watson (19) was used for precision and to produce representative images of injection sites in the figures.

## Results

### Quantitative analysis

Characteristics of the augmented breaths are represented in Fig 1. During eupnoea, augmented breaths occurred at an average frequency of one per 76.93 ± 3.7 seconds. Thus, each animal produced one augmented breath every 1–2 minutes in all preparations studied. Augmented breaths occurred spontaneously as large gasps with increases in both amplitude and duration of the various components of respiration (i.e., Ti and Te) beyond peak values of eupneic breaths. The breaths were often followed by a short period of inactivity, which was referred to as respiratory pause. The inspiration period of an augmented breath was conspicuously divided into two phases (Ti_1_ and Ti_2_). The first phase resembled typical preceding inspiratory curves and the second phase started either at the peak or near the peak of the first. The average duration of their inspiratory phase I (Ti1) was 0.19 ± 0.01 seconds while the average duration of their phase II (Ti2) was 0.15 ± 0.01 seconds (n = 30). There was a significant difference (*P* < 0.05) between Ti1 and Ti2. The average duration of the expiratory phase of an augmented breath was 0.55 ± 0.02 seconds. The amplitude of augmented breaths varied in different rats and even within the same rat. However, generally, the amplitude of the breaths ranged from about double to four times the effort of preceding inspiratory curves. The period of expiration (0.55 ± 0.02 seconds) was significantly higher than that of mean eupnoeic expiration (0.46 ± 0.01 seconds) at baseline.

### Breaths surrounding augmented breaths

Significant variation was observed in the duration of eupnoeic breathing cycles immediately after an augmented breath (Table 1), compared to those before the augmented breath. Summary values of the mean duration of inspiration and expiration of 5 breaths before (Pre AB) and after (Post AB) an augmented breath are presented in the table below.

**Table 1:** Summary of the properties of breaths immediately preceding (Pre AB) and succeeding (Post AB) an augmented breath. The Data represents the mean ± SEM of 30 representative augmented breaths.

	Ti (seconds)	Te (seconds)	RR (cycles/minute)
**Pre AB**	0.16 ± 0.004	0.33 ± 0.004	122 ± 1.3
**Post AB**	0.172 ± 0.004	0.262 ± 0.004****	141.9 ± 1.9 ****

**** *P* < 0.0001

The breaths immediately succeeding an augmented breath appeared to be shallow, with low amplitudes. The rate of these breaths (Post AB) was significantly higher than the breaths preceding an augmented breath (*P* < 0.0001). This effect resulted from a significant decrease in expiratory duration (*P* < 0.0001) and not inspiratory duration (*P* = 0.1160). However, the breaths immediately following an augmented breath had a graded increase in inspiratory amplitude until return to normal after about 5 breath cycles.

### Effects of stimulating the hippocampus on the occurrence of augmented breaths

**Fig 3:**
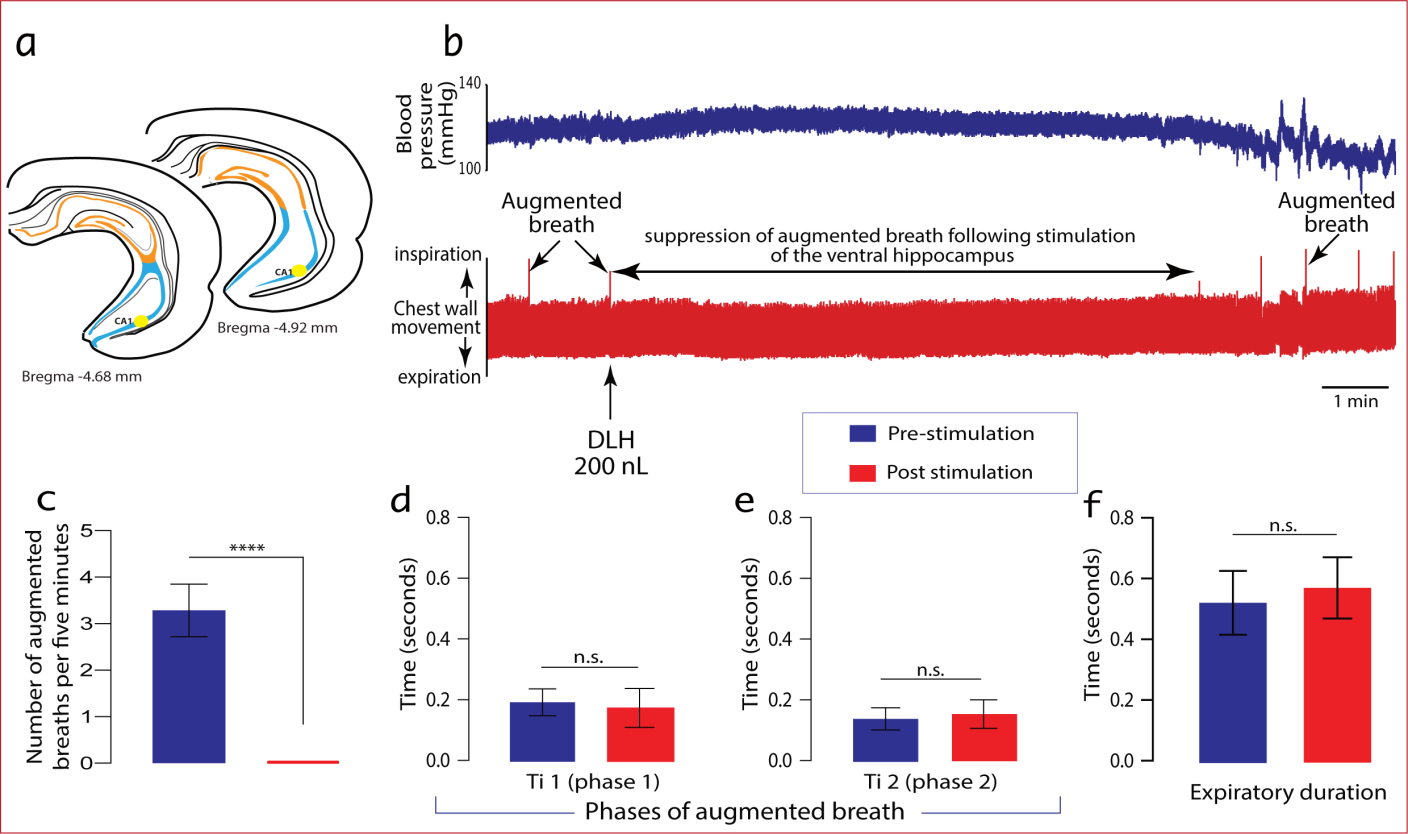
Activation of neurones in the ventral hippocampus suppress the normal periodic occurrence of augmented breaths. b) Representative tracing showing a complete suppression of augmented breaths in urethane-anaesthetised rat following an injection of 200 nL (n = 8) of DLH into the ventral CA1 region of the hippocampus (a). c) Stimulus histogram showing the frequency of occurrence of augmented breaths in the pre- and post-stimulation periods. d & e) bar charts comparing the various components of augmented breaths in the pre- and post-stimulation periods. f) Comparison of the expiratory durations before (pre) and after (post) an augmented breath.

Stimulation of the ventral hippocampus (Bregma -4.68 - -4.92 mm) caused an immediate suppression of augmented breaths (Fig 3). This effect was considered as completely suppressive and not modulatory because the general characteristics of augmented breaths pre-stimulation were identical to those post stimulation, upon resumption of the breaths. The ventral CA1 field was the specific region in the ventral hippocampus that was effective (Fig 2). The average duration of suppression was 553.7 ± 43.8 seconds (≈ 9 minutes). The dose of DLH used had no significant effect on the duration of suppression of augmented breaths. In control experiments, microinjections of normal saline made into similar areas in the ventral hippocampus failed to suppress the occurrence of augmented breaths. Stimulation of the dorsal hippocampus also has no effect on the occurrence of augmented breaths.

### Cardiorespiratory coupling during augmented breaths

The expression of augmented breaths influenced blood pressure via a transient drop in blood pressure during the peak of the second (augmented) phase of inspiration. As the augmented breath cycle progressed into the expiratory phase, blood pressure began to stabilise and only achieved stability when regular breathing cycles regain rhythmicity. This integrated cardiorespiratory effect did not change post stimulation of the ventral hippocampus.

## Discussion

There are strong correlations between the expression of emotional behaviours such as anxiety and the occurrence of augmented breaths [6, 16–18, 21]. Lesion studies in rats have showed that the ventral hippocampus is a principal limbic structure involved in expressing emotions of fear and anxiety [22]. However, the influence of the ventral hippocampus over basic physiological processes such as respiration is poorly understood. The current study is evidence that stimulation of the ventral hippocampus completely suppresses the occurrence of augmented breaths without affecting the intrinsic properties of the breaths. i.e., the augmented breaths observed before and after stimulation had characteristics that conformed with descriptions of typical augmented breaths as reported by other authors [3]. This data further suggests a link between the ventral hippocampus and respiratory rhythm generators.

The physiological implications of these findings could relate to the coupling concept of airway patency and forebrain–driven affective behaviours. Constant tidal breathing induces progressive airflow limitation, and augmented breaths can occur reflexively to improve airway patency by increasing compliance, and opening up collapsed airways [8, 23]. In fact, in mechanically ventilated preparations, performing artificial sighs (deep breaths or augmented inspiration) are important for maintaining airway patency [24]. Augmented breaths may also play a significant role in regulating airway smooth muscle tone and hence airway patency. For example, in healthy individuals, a deep breath evokes an immediate relaxation of the airway smooth muscle, a response that appears absent in asthmatics [25]. In this regard, it is intriguing that emotional (limbic) activities impact on asthma. Fear and anxiety, which are limbic-driven behaviours, are potent triggers of acute asthma exacerbation in prone individuals [26], and it is tempting to speculate that the ventral hippocampus may play a role in altering the ventilatory adjustments associated with the pathologies of respiratory conditions.

To our knowledge, the current study is the first report of the direct influence of specific neurone populations in the ventral hippocampus (Fig 3) over augmented breaths. However, a previous study had reported ventral hippocampal modulation of other components of breathing [27]. In an electrophysiology study in cats, Duffin and Hockman (1972)[27] demonstrated that electrical stimulation of the ventral hippocampus inhibits the discharge of expiratory neurones in the ventrolateral medulla, or immediately switched an expiratory phase to inspiration. This effect was also reflected in diaphragmatic discharge patterns. Although the firing pattern of ventral hippocampus neurones was not recorded and electrical stimulation with its known limitations was applied [28], the study provided a first evidence that the ventral hippocampus influences general respiratory activities. In conjunction with observations from our study, we can infer that the ventral hippocampus may constitute part of the limbic network that contributes to modulating the activities of respiratory-related cells in the ventrolateral medulla.

**Fig 4:**
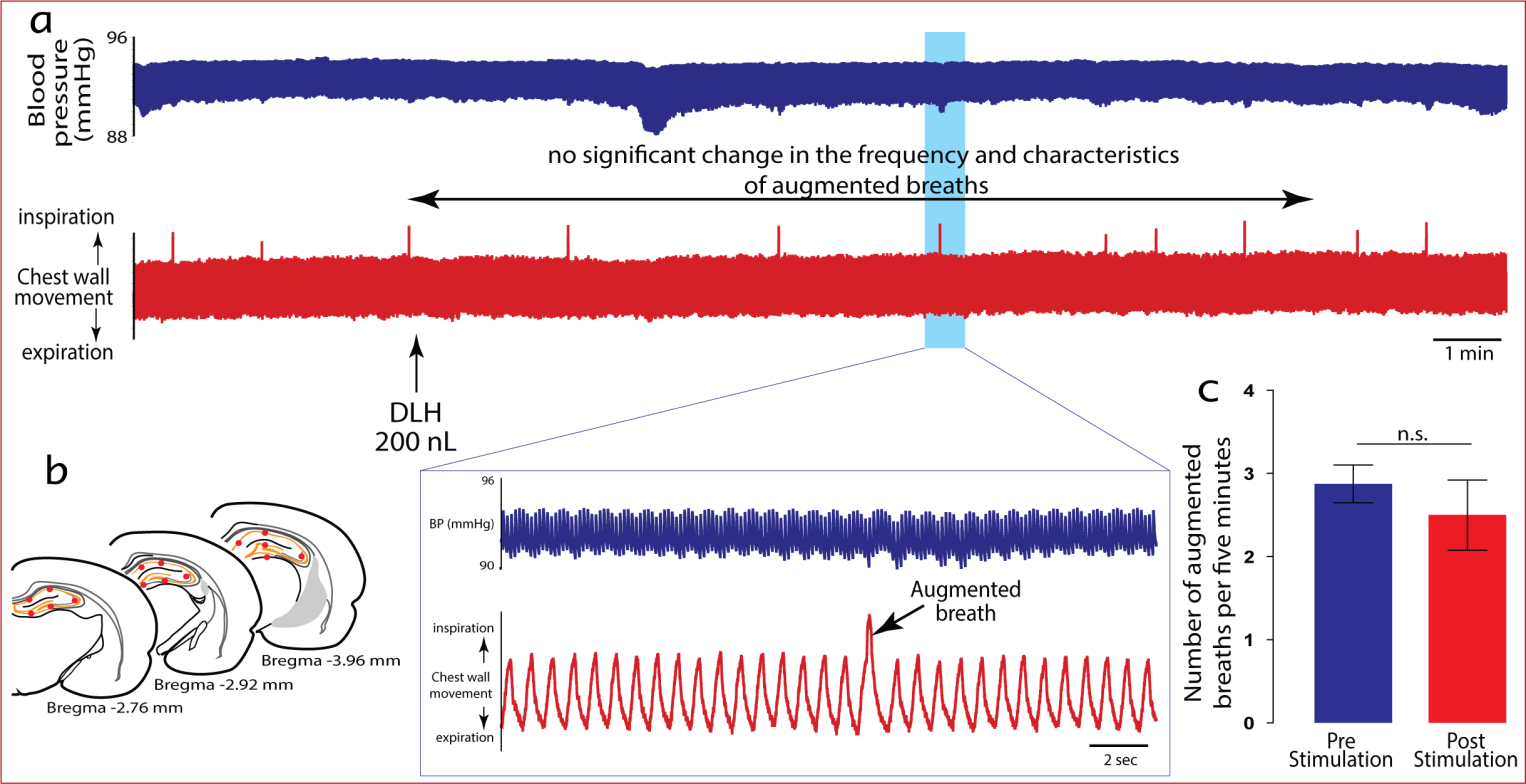
Activation of neurones in the dorsal hippocampus have no significant physiological influence over the motor expression of augmented breaths. a) Representative tracing showing a continuous motor expression of augmented breaths at relatively regular intervals in urethane-anaesthetised rat following an injection of 200 nL (n = 6) of DLH into the dorsal hippocampus (b). c) Stimulus histogram showing the frequency of occurrence of augmented breaths in the pre- and post-stimulation periods.

Interestingly, respiratory effects could not be generated from the entire hippocampus. Stimulation of dorsal hippocampal fields did not affect the frequency or expression of augmented breaths (Fig 4). This finding supports the hypothesis of functionally dissociated fields within the hippocampus [29] and suggests that the dorsal hippocampus may be involved in other functions or other aspects of emotions such as consolidation of memory.

However, previous studies have demonstrated correlations between neuronal activity in the dorsal hippocampus and the occurrence of augmented breaths in cats [30] and humans [31]. Poe et al. (1996) [30] in particular, using reflectance imaging and electrophysiological measures, demonstrated an increase in neuronal activity in the dorsal hippocampus preceding augmented breaths and during the period of respiratory pause after each augmented breath. Alternatively, studies investigating state-dependent modulation of breathing in rats have demonstrated a correlation between periods of suppressed augmented breaths and increase electroencephalogram (EEG) activity of the dorsal hippocampus [32]. In the current study, chemical micro-stimulation of the dorsal hippocampus was performed, and this did not influence the occurrence of augmented breaths. This finding suggests that activation of neurones in the dorsal hippocampus does not necessarily modulate respiratory activities but there may be some state related coupling or time lock of activity between the dorsal hippocampus and augmented breaths. However, these hypotheses must be tested by inhibition studies.

### Central modulators of augmented breaths

In reporting *in vivo* single unit recordings, studies have suggested that neurones in the preBötC region are responsible for generating augmented breaths [4, 5]. Further studies have demonstrated modulatory effects of higher brain structures, such as the hypothalamus [15] and periaqueductal gray [33], on the activities of augmented breaths. With the amygdala being a key limbic structure that recruits subcortical and brainstem structures for complete emotional expression, it is conceivable that it may also modulate augmented breaths. This inference conforms with the previous findings that have reported increased frequency of augmented breaths in subjects with a history of anxiety disorders [34]. It also fits the hypothesis that negative emotions (e.g., fear, anxiety and panic) and positive emotions (e.g., pleasure and relief) strongly influence the occurrence of augmented breaths [6]. Perhaps there is a physiological interaction between the ventral hippocampus and amygdala that regulates the occurrence of augmented breaths.

### Link between behaviour and augmented breaths

Limbic regulation of the occurrence of augmented breaths may not be confined to the hippocampus. Two chemical microinjection studies have shown that disinhibition of the dorsomedial hypothalamus and stimulation of the dorsal PAG influence the onset and frequency of augmented breaths [15, 33]. These regions play key roles in appropriating behaviours that reflect the state of an animal. For instance, the dorsomedial hypothalamus maintains the sleep-wake circadian rhythm [35] and there may be a link between such state-transition and the expression augmented breaths [6]. Alternatively, the PAG contains discrete neuronal populations and circuits that control a range of emotional behaviours [7, 36], thus permitting a ‘switch’ from one emotional state to another. The ability to influence transitions between emotional states may explain why the PAG affects the expression of augmented breaths [6]. Also, connections between the PAG and respiratory-related neurones in the ventrolateral medulla have been established [37, 38]. It is important to note that the activities of both the hypothalamus and PAG are subject to modulation from higher limbic areas [39], one of which could be the ventral hippocampus.

### Cardiorespiratory characteristics of augmented breaths

Augmented breaths are associated with characteristic changes in blood pressure and heart rate. The mechanisms supporting the coupled cardiorespiratory dynamics as observed in the current study can be explained in the light of the influence of changes in intra-thoracic/abdominal pressure over venous return [40]. Intra-thoracic pressure has a strong relationship with pressure in the right atrium. During inspiration, the thoracic cavity expands and creates a negative pressure in the thoracic cavity and pleural spaces. Consequently, the pressure in the vena cava, pulmonary vessels and capillaries decrease. As a result of these differences in pressures, there is a brief fluctuation in blood pressure. More so, during the augmented phase of breathing the abdominal muscles are recruited due to the extensive downward movement of the diaphragm [41], which increases intra-abdominal pressure. This effect is, in turn, capable of increasing pressure in the abdominal portion of the vena cava and the return of venous blood to the right atria. These respiratory mechanical influences account for the brief spike of blood pressure as seen in our study (Fig 3). The physiological dynamics occurring with blood pressure, as observed at the end of trace B, Fig 3, was not clear but was considered an artefact and so was not discussed. However, it is possible that a reset in cardiorespiratory activities could have led to a period of brief dysrhythmia.

In addition to the cardiorespiratory coupling, the breaths surrounding each augmented breath had peculiar characteristics. In the current study, low amplitudes in the first few breaths after an augmented breath (between 1–5 breaths) were typical but with a gradual return to normal breathing cycles after approximately 4–5 breathes. This consistent characteristic is similar to augmented breaths that were evoked by stimulating peripheral chemoreceptors using almitrine [42]. In the case of almitrine-induced augmented breaths, post-augmented breaths were characterised by diminished airflow. The reduced amplitudes of inspiration observed in the current study imply reduced airflow.

### Effects of urethane anaesthesia on augmented breaths

Studies investigating the frequency of occurrence of augmented breaths under various physiological states and commonly used injectable anaesthetics have suggested that urethane anaesthesia at a dose of 1.2 g/kg completely suppresses the occurrence of augmented breaths in rats spontaneously breathing room air [11]. The current study is empirical evidence of the regular and frequent occurrence of augmented breaths in urethane-anaesthetised rats spontaneously breathing room air. In fact, doses as high as 1.5 g/kg did not produce any changes in the general characteristics of augmented breaths. Similar doses have also aided investigations of augmented breath phase volume and timing relationship, and state-dependent modulation in rats [3, 32]. In the current investigation, urethane anaesthesia produced stable respiratory and cardiovascular parameters, with a regular and consistent occurrence of augmented breaths, for prolonged periods without additional pharmacological manipulations. These characteristics also suggest stable firing of respiratory-related neurones as demonstrated by previous authors [43].

### Study limitations

Given the influence of brain state on breathing function and sigh rate [44], an attempt to concomitantly record EEG activity from the hippocampus during stimulation, to determine the overall effect of DLH on hippocampal activity, could strengthen the current findings. However, traditional EEG protocols, which are typically non-invasive with electrodes placed on the scalp, was not accommodated because of the technical difficulties associated with the current experimental setup particularly the provision of clear scalp space for stereotaxic navigation of micropipettes without impediments. Future studies may also consider simultaneous extracellular recordings with high impedance electrodes to produce fine focal resolutions of neuronal activity in the stimulated area.

### Conclusion

In conclusion, the data supports the hypothesis that there is a functional relationship between neurones of the ventral hippocampus and brainstem nuclei that control augmented breaths. This relationship may support the regulation of specific components of respiration during emotional challenges.
