# Correction: Effects of the hippocampus on the motor expression of augmented breaths

**DOI:** 10.1371/journal.pone.0220760

**Published:** 2019-08-01

**Authors:** Itopa E. Ajayi, Paul C. Mills

With this notice, the authors provide an updated Data Availability Statement and underlying data supporting the results in this article [1].

The updated Data Availability Statement is: The underlying data supporting the results of this article [1] are provided with this Correction notice, its Supporting Information files and at https://figshare.com/s/c820fbf3bf144f900621.

The Figshare dataset includes raw tracing data files for experiments involving neurochemical stimulation of the ventral hippocampus (n = 8), stimulation of the dorsal hippocampus (n = 6), and control animals that received vehicle-only stimulation (n = 2), as described in Tables 1–3 of this notice. Each file was from a separate animal with the exception of R83 and R83b.

The authors apologize for not including the underlying data with the original article.

**Table 1:** Raw tracing Data files represents experiments for the neurochemical microstimulation of the ventral hippocampus (n = 8). Each file was from a separate animal with the exception of R83 and R83b where there was a software glitch that caused us to restart the software. Hence the reason for the labelling.

S/N	File Name	Brief description of the content
1.	R55	Stimulation of the ventral hippocampus to check for any changes to the occurrence of augmented breaths. The results showed a suppression of augmented breaths.
2.	R57	Stimulation of the ventral hippocampus to check for any changes to the occurrence of augmented breaths. The results showed a suppression of augmented breaths.
3.	R59	Stimulation of the ventral hippocampus to check for any changes to the occurrence of augmented breaths. The results showed a suppression of augmented breaths.
4.	R62	Stimulation of the ventral hippocampus to check for any changes to the occurrence of augmented breaths. The results showed a suppression of augmented breaths.
5.	R73	Stimulation of the ventral hippocampus to check for any changes to the occurrence of augmented breaths. The results showed a suppression of augmented breaths.
6.	R76	Stimulation of the ventral hippocampus to check for any changes to the occurrence of augmented breaths. The results showed a suppression of augmented breaths.
7.	R82	Stimulation of the ventral hippocampus to check for any changes to the occurrence of augmented breaths. The results showed a suppression of augmented breaths.
8.	R83	Stimulation of the ventral hippocampus to check for any changes to the occurrence of augmented breaths. The results showed a suppression of augmented breaths.
9.	R83b	Stimulation of the ventral hippocampus to check for any changes to the occurrence of augmented breaths. The results showed a suppression of augmented breaths.

**Table 2:** Raw tracing Data files for the neurochemical microstimulation of the dorsal hippocampus (n = 6). Each file represents a separate animal.

S/N	File Name	Brief description of the content
1.	R54	Stimulation of the ventral hippocampus to check for any changes to the occurrence of augmented breaths. The results showed a suppression of augmented breaths.
2.	R63	Stimulation of the ventral hippocampus to check for any changes to the occurrence of augmented breaths. The results showed a suppression of augmented breaths.
3.	R67	Stimulation of the ventral hippocampus to check for any changes to the occurrence of augmented breaths. The results showed a suppression of augmented breaths.
4.	R69	Stimulation of the ventral hippocampus to check for any changes to the occurrence of augmented breaths. The results showed a suppression of augmented breaths.
5.	R70	Stimulation of the ventral hippocampus to check for any changes to the occurrence of augmented breaths. The results showed a suppression of augmented breaths.
6.	R75	Stimulation of the ventral hippocampus to check for any changes to the occurrence of augmented breaths. The results showed a suppression of augmented breaths.

**Table 3:** Raw tracing Data files for the Vehicle injection into the ventral hippocampus (n = 2). Each file represents a separate animal.

S/N	File name	Brief description of content
1.	R91	Microinjection of the ventral hippocampus with a normal saline to check for any systemic changes in the breathing and the motor expression of augmented breaths.
2.	R93	Microinjection of the ventral hippocampus with a normal saline to check for any systemic changes in the breathing and the motor expression of augmented breaths.

## Supporting information

S1 File Photomicrograph of the injection site into the ventral hippocampus of R55. (JPG)

S2 File Photomicrograph of the injection site into the ventral hippocampus of R57. (JPG)

S3 File Photomicrograph of the injection site into the ventral hippocampus of R59. (JPG)

S4 File Photomicrograph of the injection site into the ventral hippocampus of R62. (JPG)

S5 File Photomicrograph of the injection site into the ventral hippocampus of R73. (JPG)

S6 File Photomicrograph of the injection site into the ventral hippocampus of R76. (JPG)

S7 File Photomicrograph of the injection site into the ventral hippocampus of R82. (JPG)

S8 File Photomicrograph of the injection site into the ventral hippocampus of R83. (JPG)

S9 File Photomicrograph of the injection site into the dorsal hippocampus of R54. (JPG)

S10 File Photomicrograph of the injection site into the dorsal hippocampus of R63. (JPG)

S11 File Photomicrograph of the injection site into the dorsal hippocampus of R67. (JPG)

S12 File This is a Microsoft Excel document that contains the all the raw data for the experiments conducted. (XLSX)

S13 File This Graphpad Prism file contains an analysis of the eupneic breaths that surround an augmented breath. The file contains the inspiratory and expiratory durations of the surrounding eupneic breaths.
(PZFX)

S14 File This Graphpad Prism file contains a raw analysis of the components of augmented breaths. The file contains the durations of all the components of an augmented breath.
(PZFX)

S15 File This Graphpad Prism file contains a comparative analysis augmented breaths pre and post neurochemical microstimulation. (PZFX)

S16 File This Graphpad Prism file contains an analysis of the frequency of augmented breaths in the dorsal hippocampus. (PZFX)

S17 File This Graphpad Prism file contains an analysis of the frequency of augmented breaths in the ventral hippocampus. (PZFX)

S18 File This file contains an analysis of the intervals in the occurrence of normal augmented breaths i.e. the interval between normal augmented breaths. (PZFX)
