# Supplementary figures and images for: Correction: Effects of the hippocampus on the motor expression of augmented breaths
Source: PLoS One. 2019 Aug 1;14(8):e0220760. doi: 10.1371/journal.pone.0220760 (PMC6675070; doi:10.1371/journal.pone.0220760)

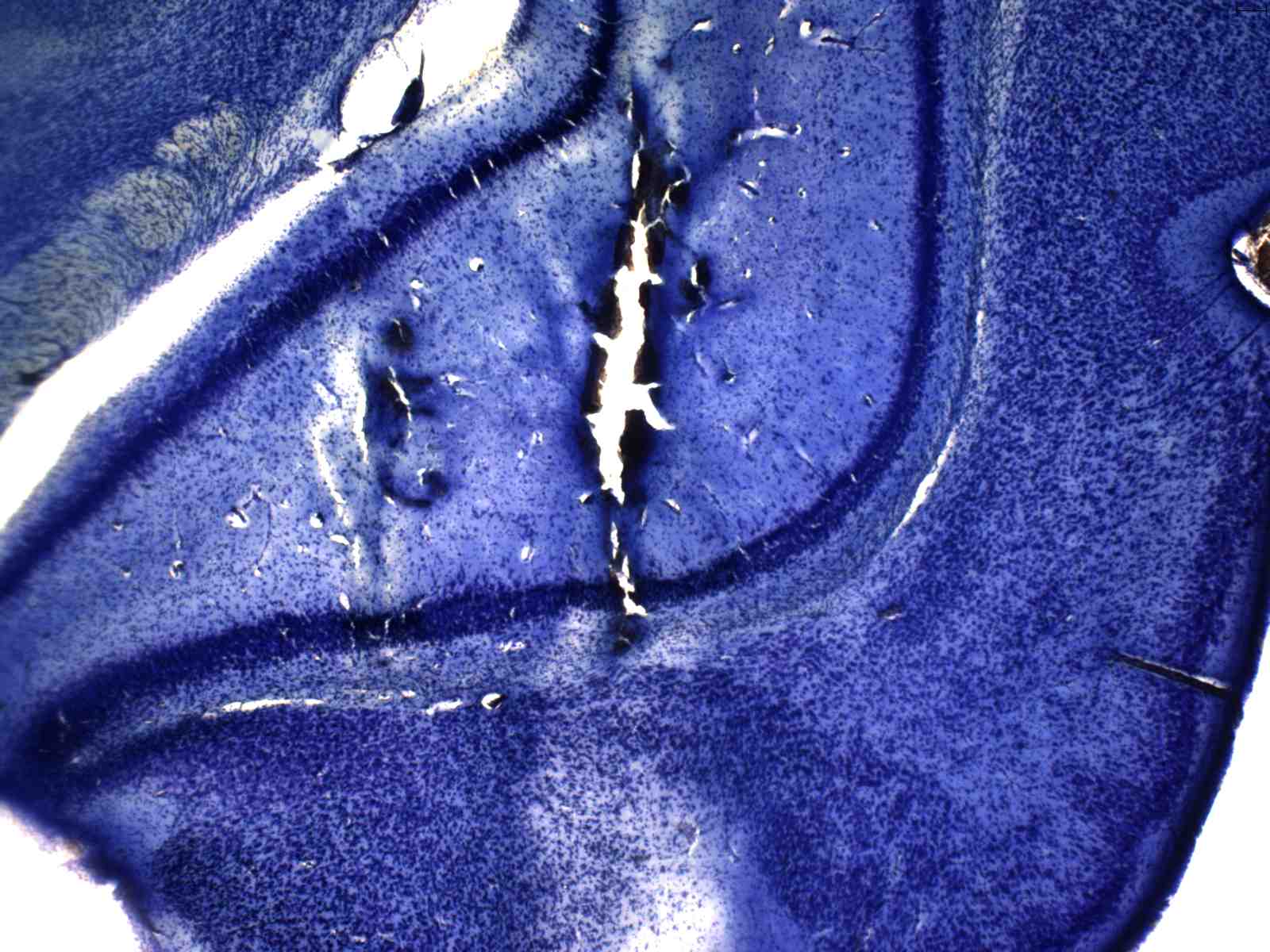

Supplement: S1 File — (JPG) [file pone.0220760.s001.jpg]

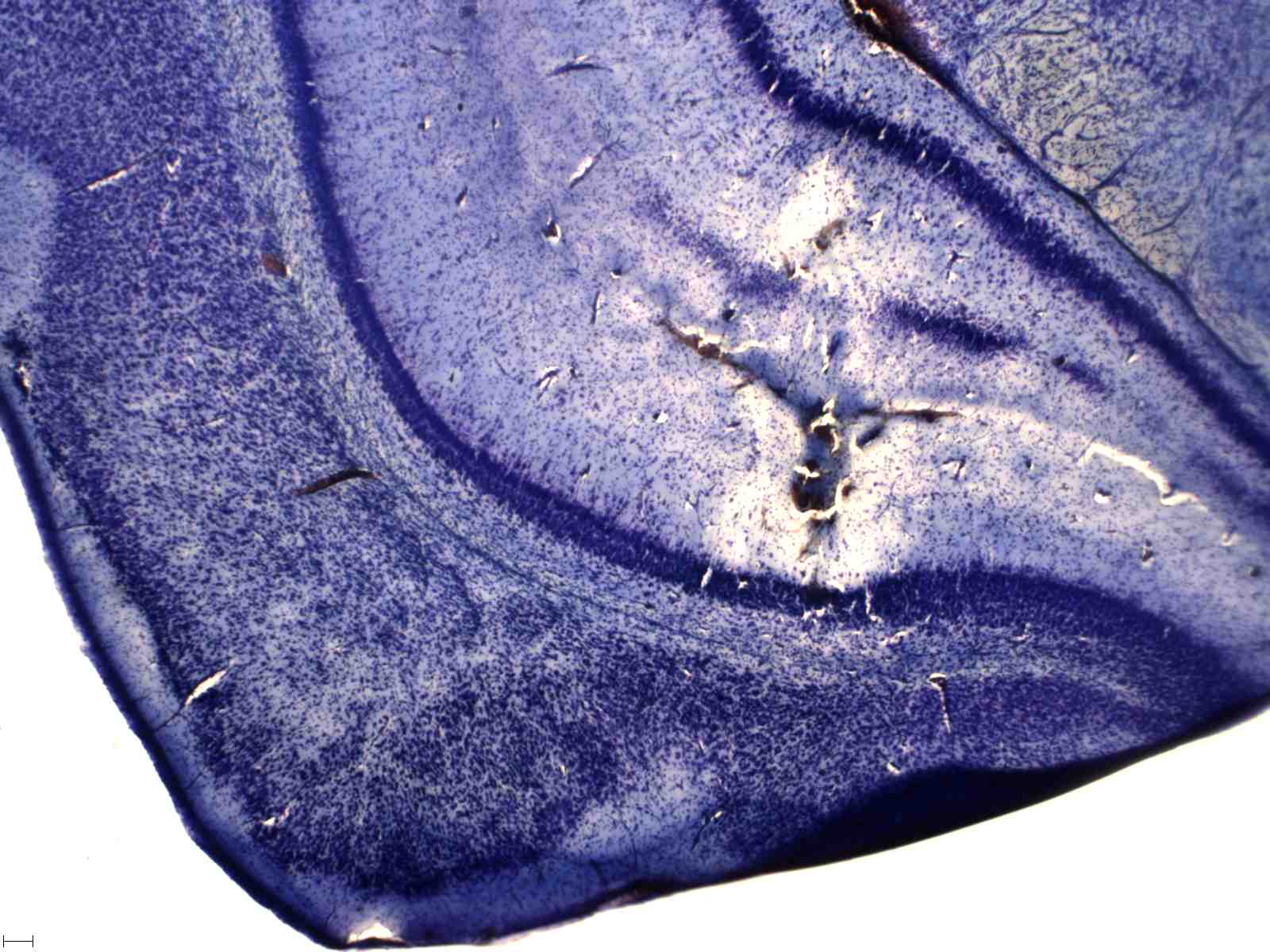

Supplement: S2 File — (JPG) [file pone.0220760.s002.jpg]

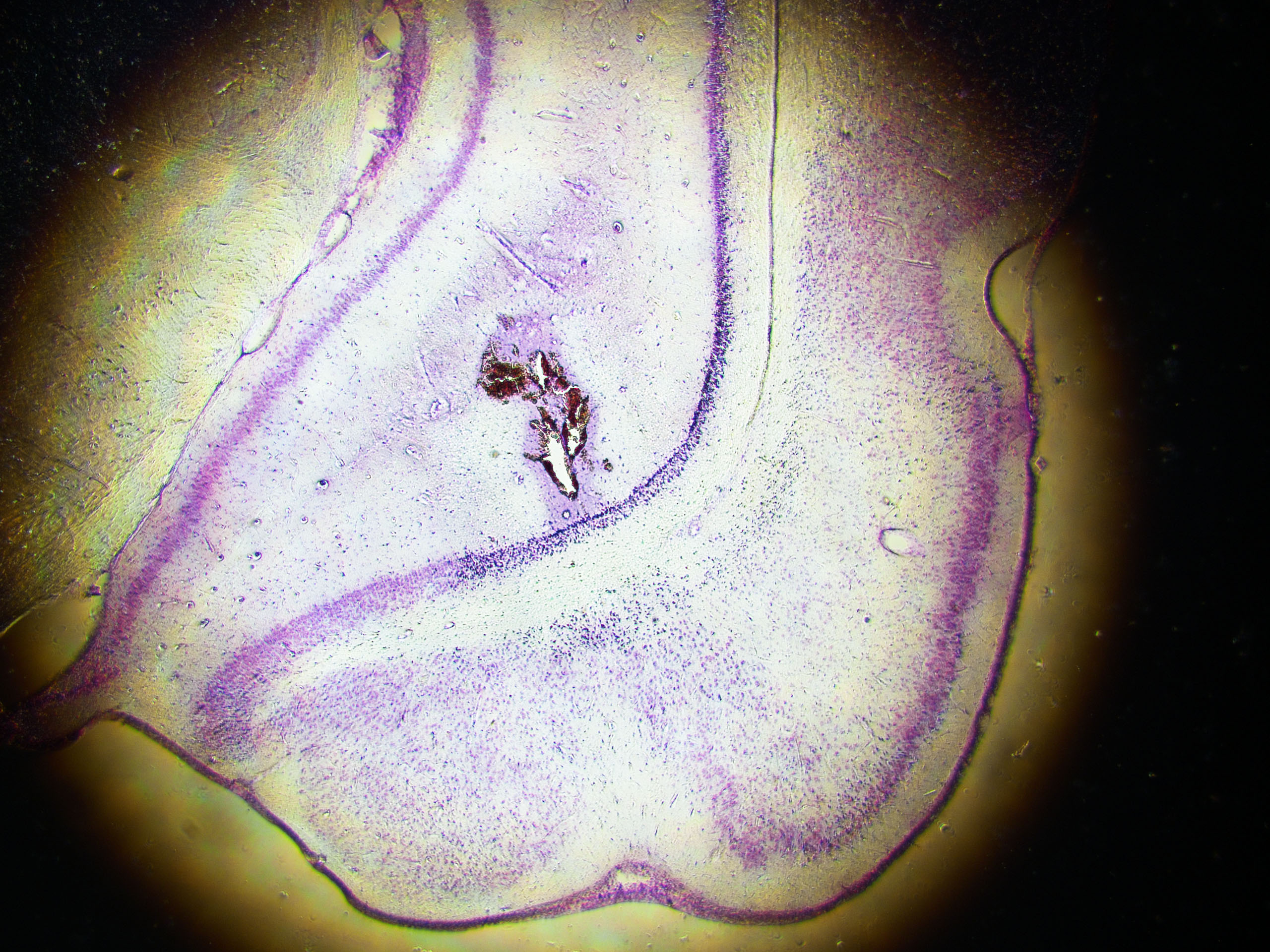

Supplement: S3 File — (JPG) [file pone.0220760.s003.jpg]

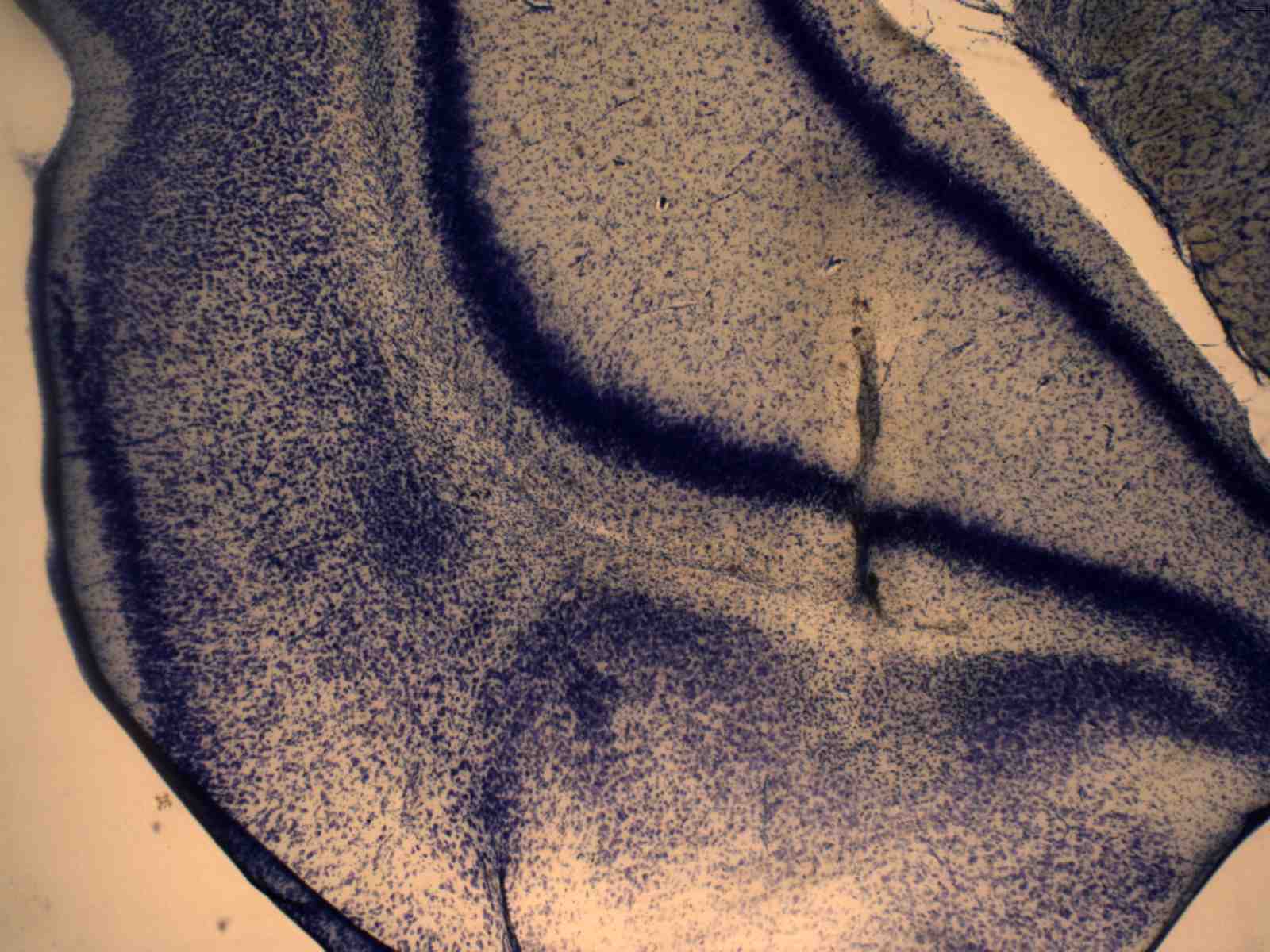

Supplement: S4 File — (JPG) [file pone.0220760.s004.jpg]

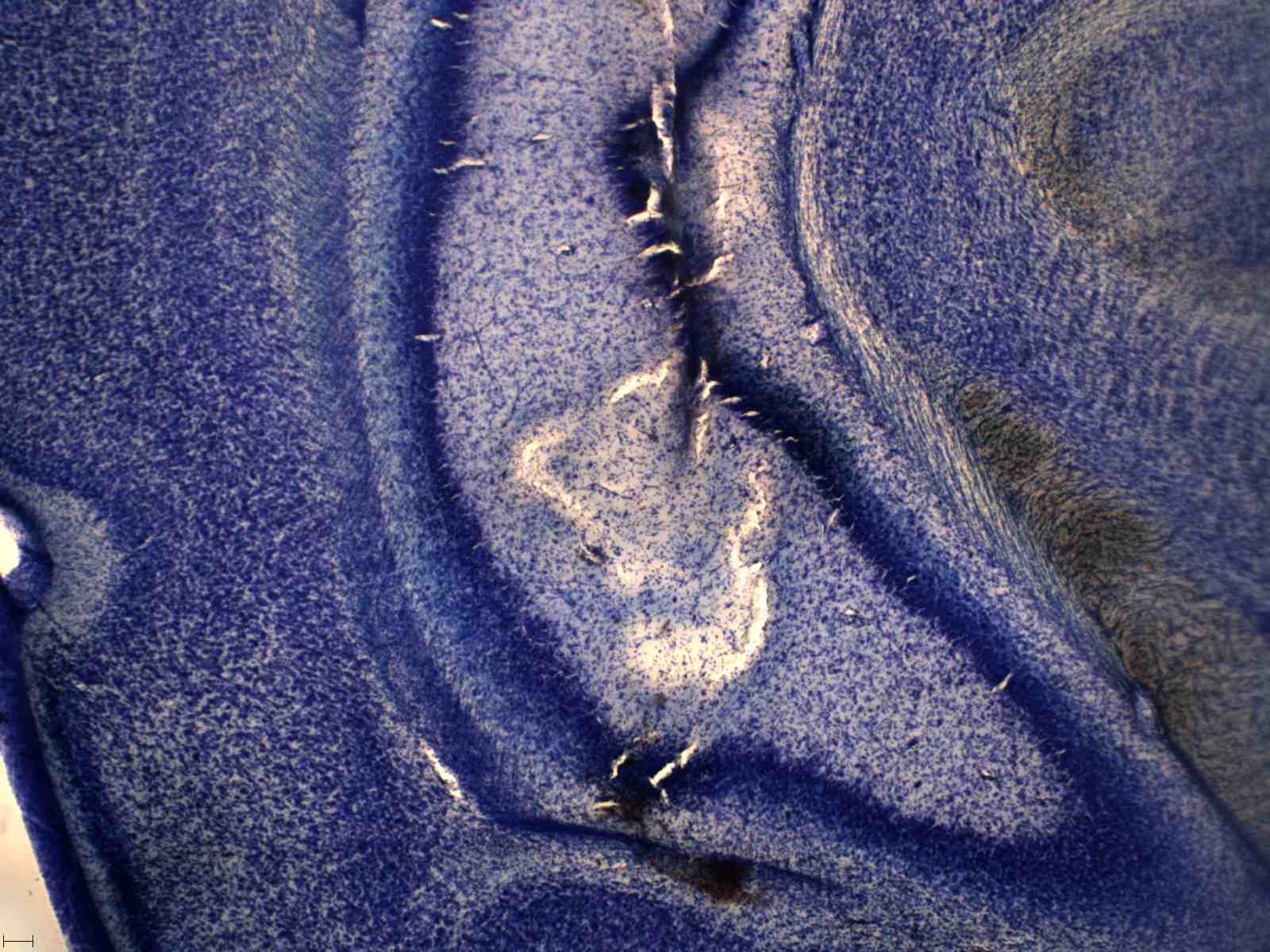

Supplement: S5 File — (JPG) [file pone.0220760.s005.jpg]

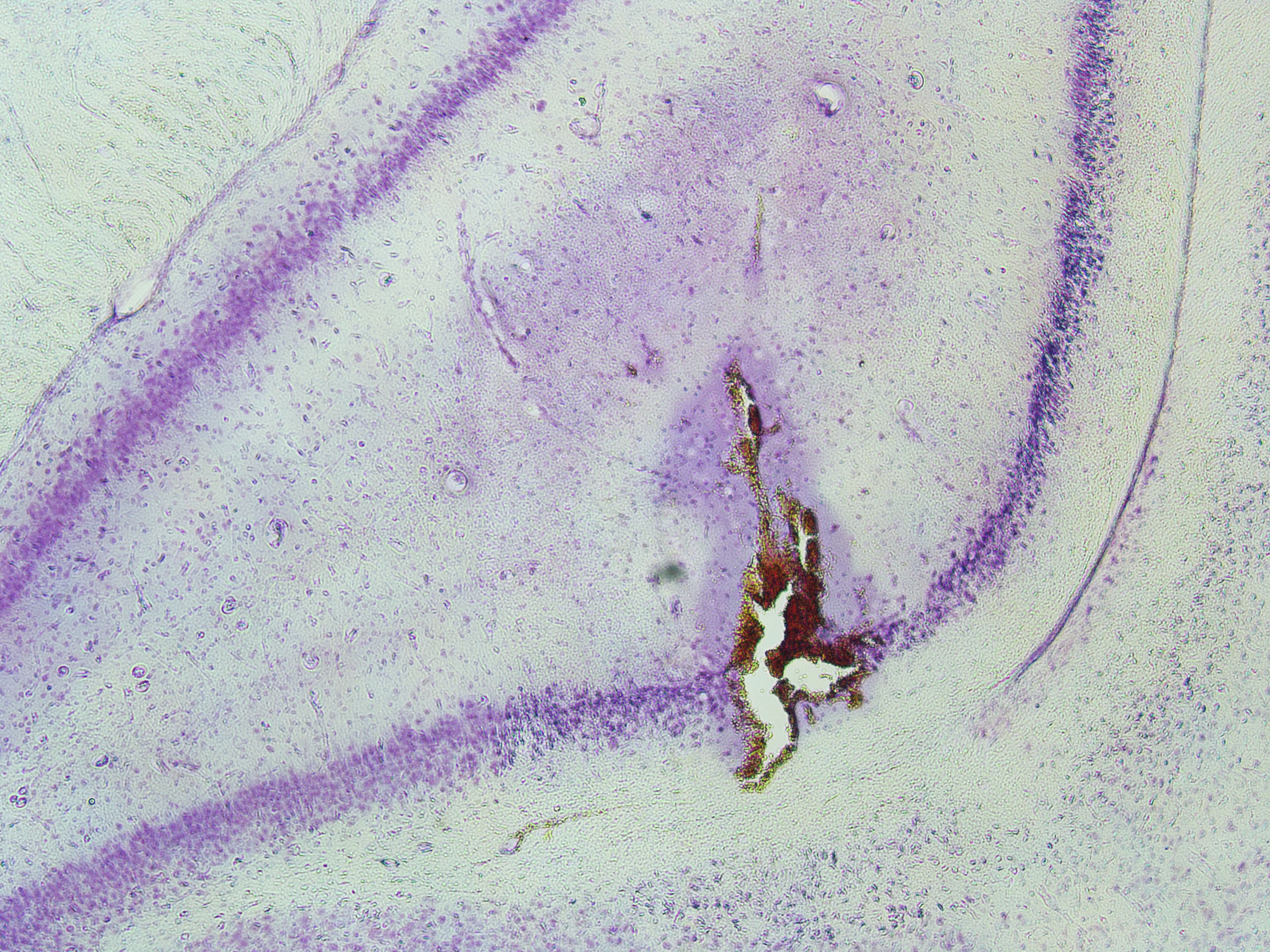

Supplement: S6 File — (JPG) [file pone.0220760.s006.jpg]

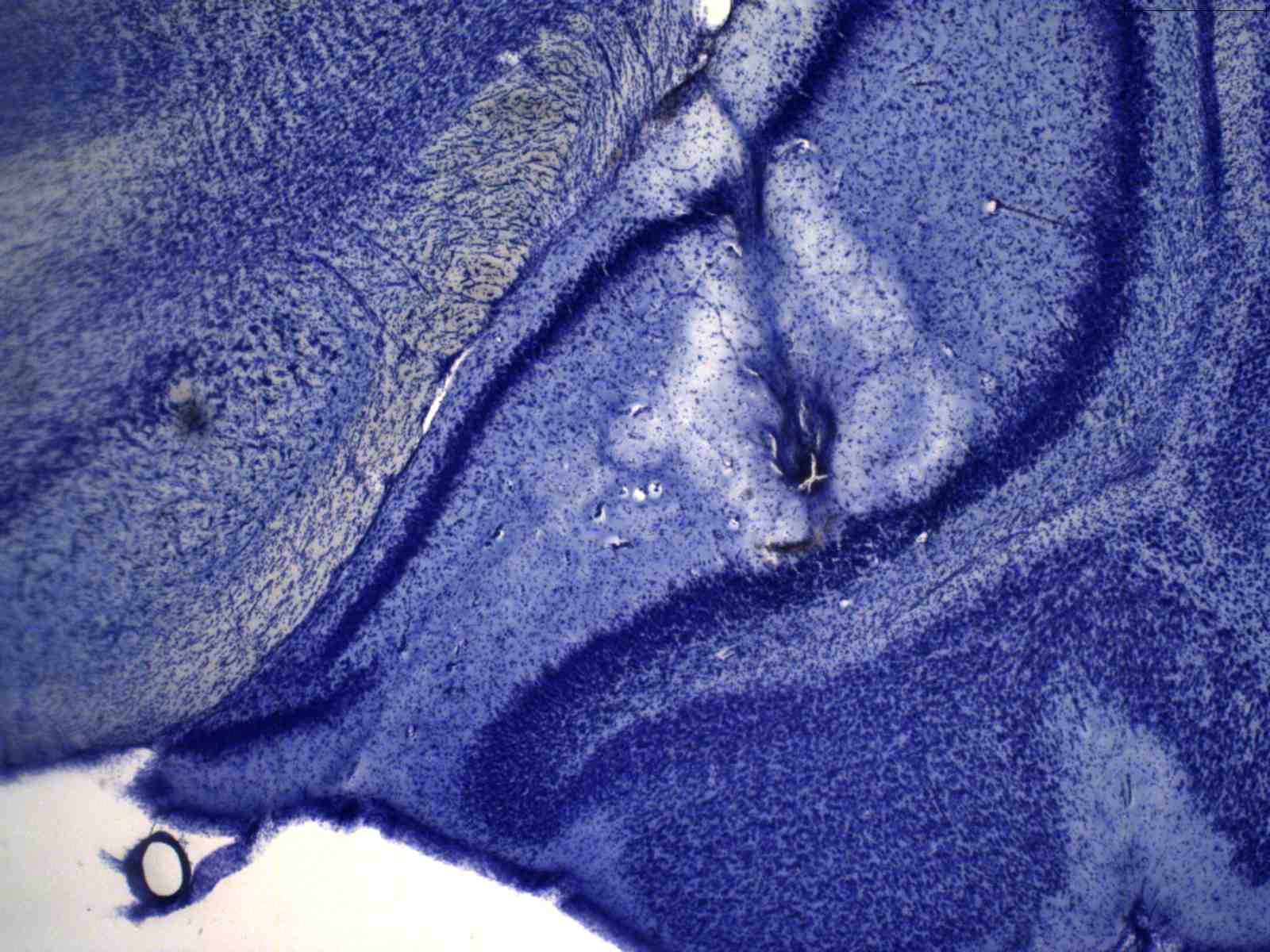

Supplement: S7 File — (JPG) [file pone.0220760.s007.jpg]

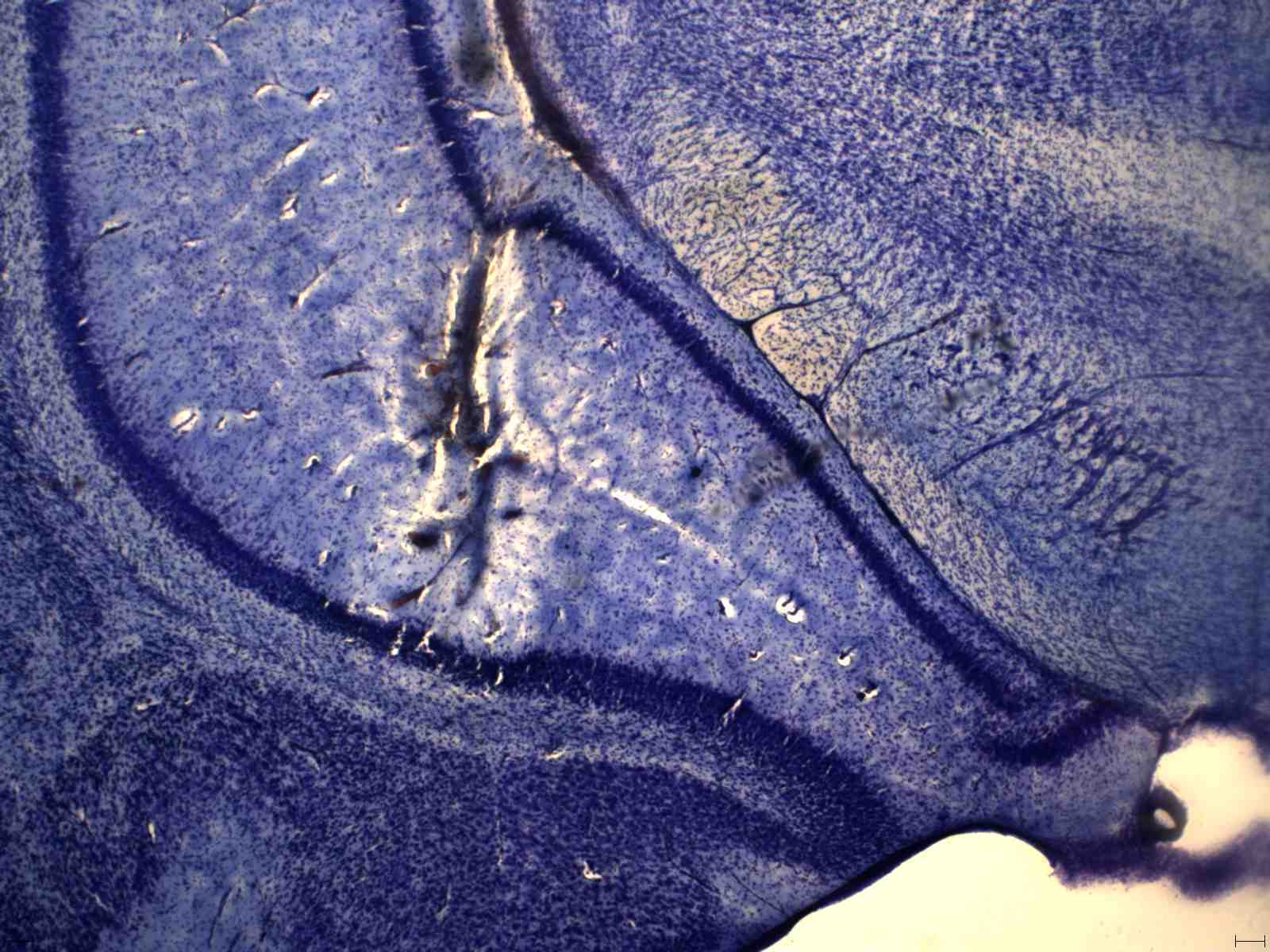

Supplement: S8 File — (JPG) [file pone.0220760.s008.jpg]

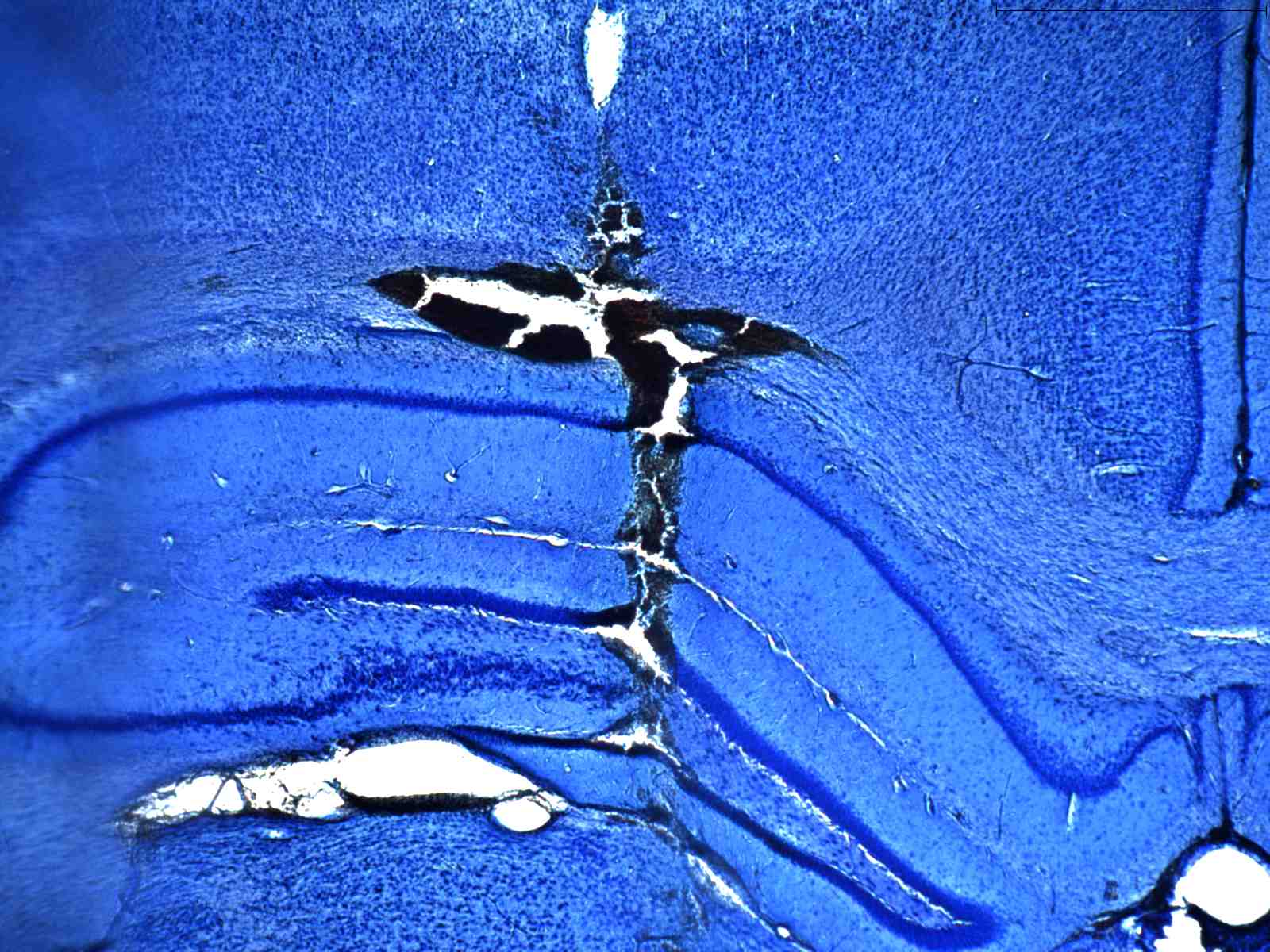

Supplement: S9 File — (JPG) [file pone.0220760.s009.jpg]

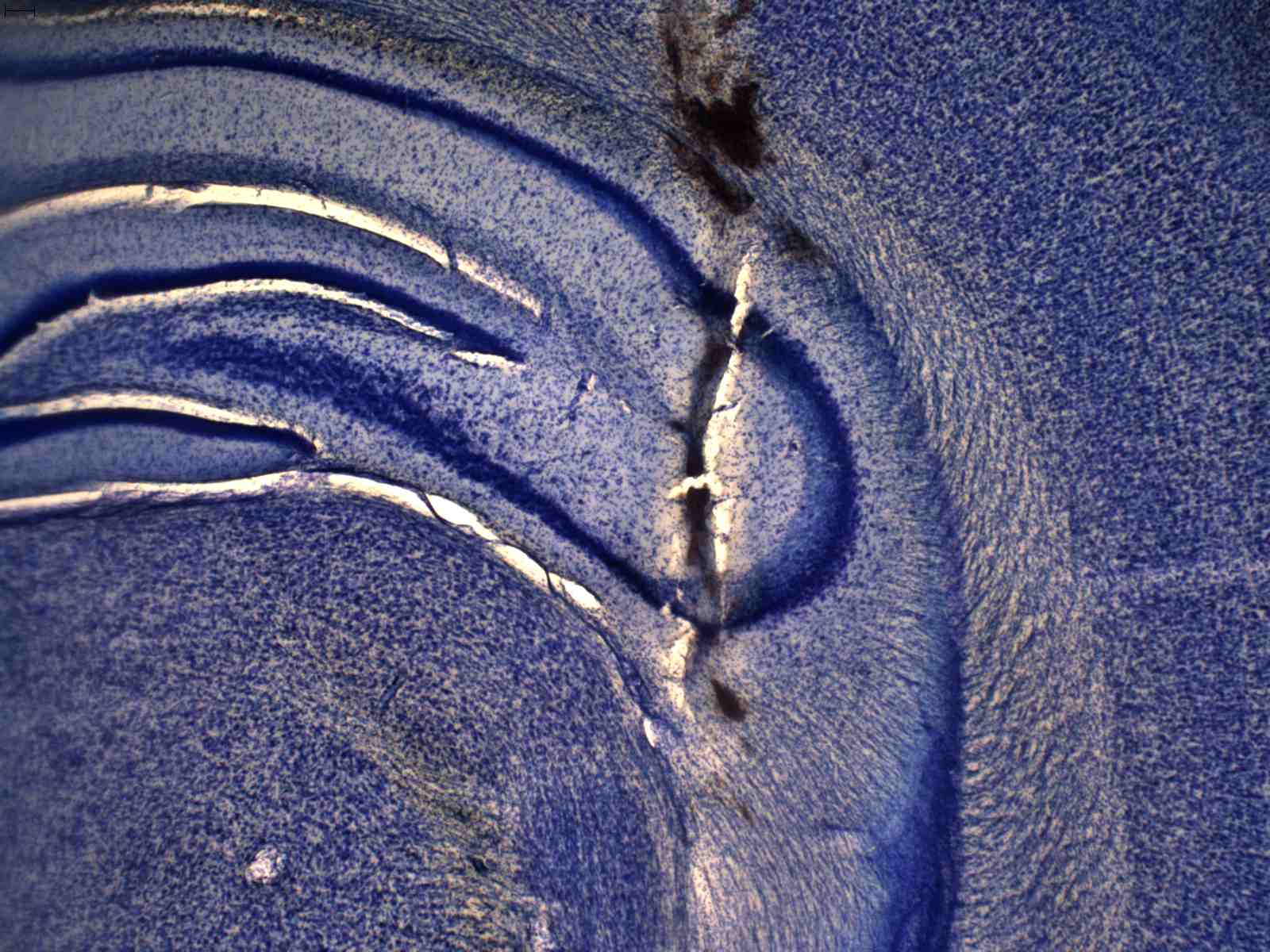

Supplement: S10 File — (JPG) [file pone.0220760.s010.jpg]

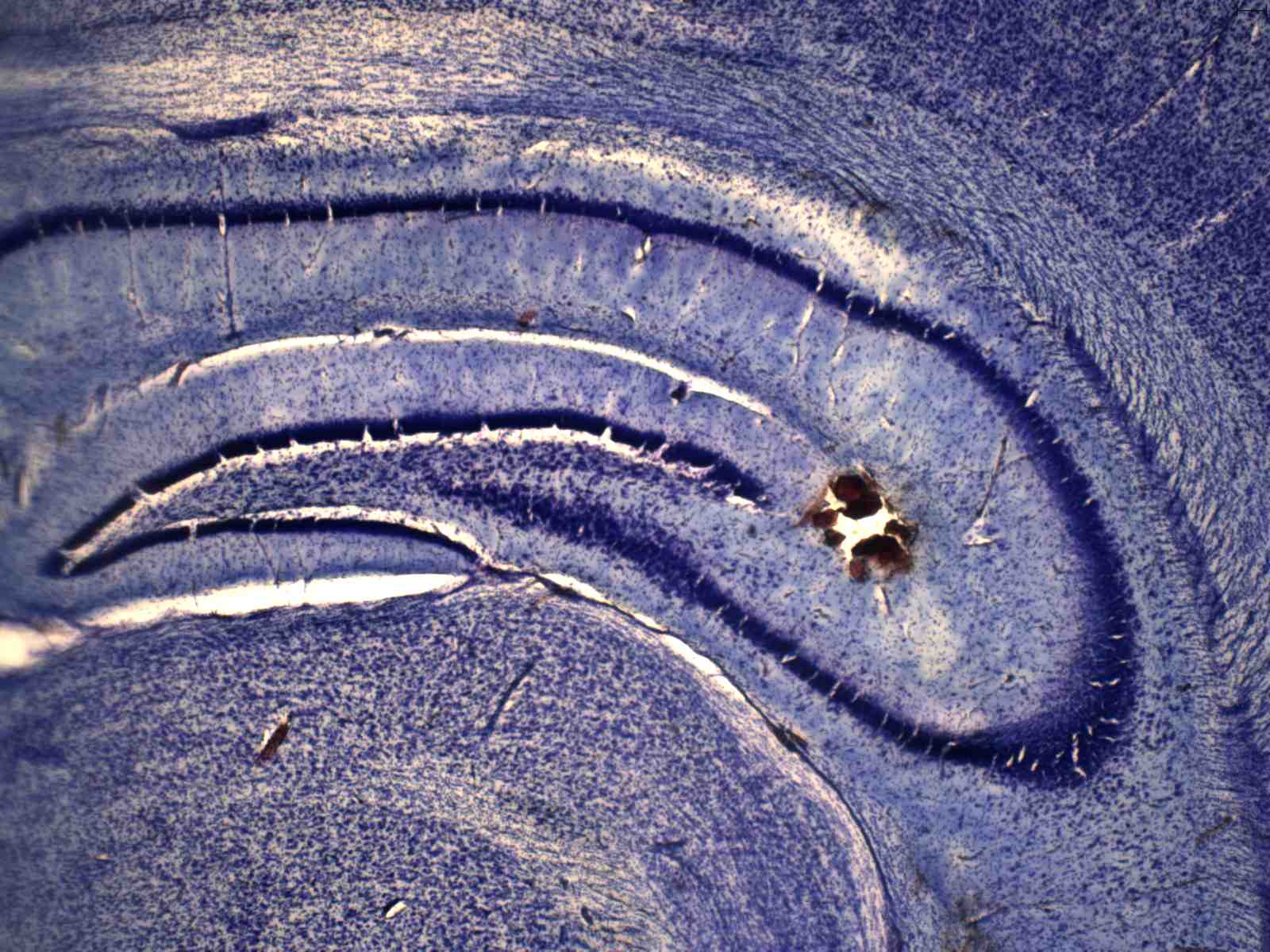

Supplement: S11 File — (JPG) [file pone.0220760.s011.jpg]
